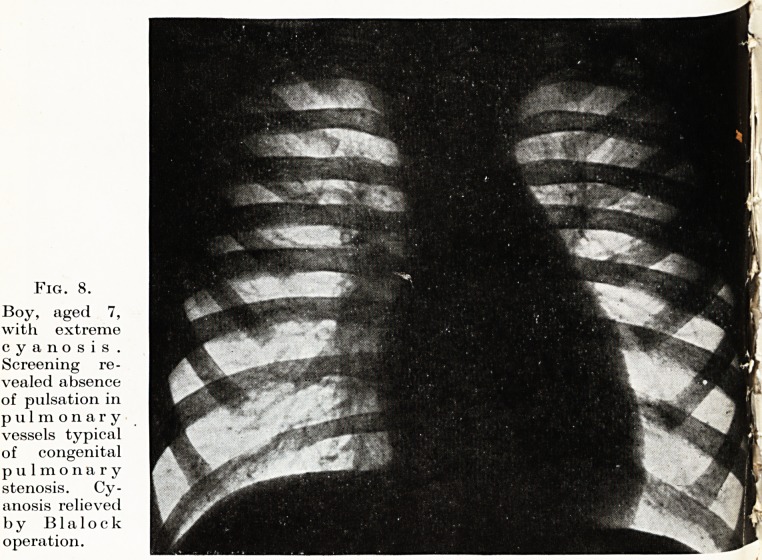# The Diagnostic Work of a Surgical Thoracic Unit, with Special Reference to Intra-Thoracic Suppuration and Bronchogenic Carcinoma
*An address to the Bristol Division of the British Medical Association on 21st May, 1947.


**Published:** 1948

**Authors:** R. H. R. Belsey

**Affiliations:** Special Lecturer in Thoracic Surgery, University of Bristol


### THE DIAGNOSTIC WORK OF A SURGICAL THORACIC UNIT, WITH SPECIAL REFERENCE TO INTRA-THORACIC SUPPURATION AND BRONCHOGENIC CARCINOMA.*

BY

R. H. R. Belsey, M.B., M.S., F.R.C.S.
*Special Lecturer in Thoracic Surgery, University of Bristol.*

---

A survey of the work of Thoracic Units since 1939 reveals an increasing proportion of purely diagnostic work. Of the five thousand cases that have passed through the Unit at Kewstoke and Frenchay since 1942 approximately one half were cases of obscure chest disease referred for investigation, irrespective of whether the ultimate treatment might be operative or non-operative. It is interesting to study the stages by which the diagnostic methods essential to the study of chest diseases have been adopted and modified by the chest surgeon.

*Clinical examination.*—With increasing experience more and more reliance is placed on auscultation and the other simple methods of examining the chest ; secondly, no examination of the chest is complete without a careful examination of the neck and upper abdomen. The thoracic surgeon is in a fortunate position regarding the interpretation of physical signs by virtue of the unique opportunity of correlating clinical findings with the results of the special investigations, about to be outlined, on the relatively vast amount of clinical material that gravitates to a thoracic unit. Only by the full employment of all methods of investigation now available and the integration of the evidence so obtained can accuracy in diagnosis be achieved.

*Sputum examination.*—All that was taught by the master clinicians of the nineteenth century concerning naked eye appearances of sputum is valid to-day. Sputum culture is being replaced by examination of secretion obtained from the periphery of the lung, either through the bronchoscope, or by other methods to be described presently. Sputum examination for cancer cells has only limited application ; the technique is tedious and there are few cases where diagnosis of malignant disease cannot be made by simpler measures less open to misinterpretation.

* An address to the Bristol Division of the British Medical Association on 21st May, 1947.

*Endoscopy.*—An important event in the history of diagnostic method was the invention of the bronchoscope and oesophagoscope by the throat surgeons, who for many years remained the only clinicians versed in the use of these instruments. But with the advent of pneumonectomy in the treatment of cancer of the lung the chest surgeon needed information upon the possible spread of the growth, which the laryngologist, who saw only the endobronchial side of the neoplasm, was unable to offer. The thoracic surgeon on the other hand, aided by the opportunity of correlating thoracotomy and bronchoscopic findings, has been able to acquire considerable experience in the interpretation of the more subtle endoscopic signs of the spread of the disease towards the mediastinum. The written or verbal report can never convey as much as the vivid impression gained by the operator performing the examination.

Bronchoscopy has contributed much to our knowledge of the pathology of chest disease, especially in relation to pulmonary tuberculosis and hilar lymphadenitis in childhood ; the incidence of bronchial ulceration and broncho-stenosis in adults ; the pathology of the bronchogenic tumours, especially the benign tumours ; the influence of broncho-stenosis in the aetiology of bronchiectasis ; the localization of lung abscesses. Bronchoscopy has become a routine step in the investigation of the majority of chest cases, and repeated bronchoscopy may reveal lesions not visible at the first examination. Whenever a lipiodol bronchogram is indicated, so also is bronchoscopy. (Fig. 1.)

Surgery of the oesophagus is advancing rapidly and an increasing number of operations upon this organ are being performed in the thoracic units. Not least of the factors contributing to this progress is the increasing use of the oesophagoscope. Again, it is the more subtle findings that are of the greatest importance. Early carcinoma of the oesophagus can only be discovered on oesophagoscopy ; a barium swallow is frequently negative. (Fig. 2.)

*Thoracoscopy* following the induction of a diagnostic pneumothorax is occasionally of value in the diagnosis of lesions of the pleura and mediastinum.

*Paracentesis.*—Exploration of the chest with a needle can be of utmost importance. In fact, it provides us with the only positive physical sign of an empyema, pus in the aspirating syringe, but it is a procedure which may lead to the most dire consequences, and should only be undertaken with extreme caution. Tragedies have occurred following the injudicious use of the needle, and ideally, thoracocentesis should never be undertaken until an antero-posterior and lateral X-ray of the chest have been examined. Under no circumstances should any attempt be made to aspirate a lung abscess. The differential diagnosis between a lung abscess and an empyema or sub-phrenic abscess, which the patient is coughing up through a bronchial fistula, is extremely difficult at times, even with the help of X-rays. Any attempt to needle a sub-phrenic abscess is also to be deprecated, as an empyema usually results. In needling a chest the common mistake of going too low should be avoided, and on no account should air be admitted to the chest through the needle. If pus is found it is inadvisable to inject penicillin until the fluid has been cultured and the diagnosis completed.

*Biopsy* examination of tissues removed from within the chest is playing an increasingly important role in the diagnosis of intra-thoracic disease. There are two methods of obtaining biopsies from the lung itself: the "punch" method, in which material is obtained by aspiration through a wide-bore needle, and the open method where a portion of the rib is resected under local anaesthesia, and the material obtained under direct vision. Of the two, the latter method is probably the safer and more informative. Whenever a collection of pus is drained, a portion of the wall of the abscess is removed for histological examination, which may be the only method of establishing a diagnosis of tuberculosis or actinomycosis. (Fig. 3.)

*Exploratory thoracotomy* is now established as an essential step in the early diagnosis of carcinoma of the lung, and may be the only method of confirming the diagnosis at a stage when no direct or indirect evidence of malignancy is obtainable on bronchoscopy, when X-ray appearances are equivocal, and abnormal physical signs are entirely absent—the only stage at which the growth may be operable. It is not a severe operation and involves less risk to the patient than an exploratory laparotomy. There is a growing conviction, based on thoracotomy evidence, that cancer of the lung may remain a strictly localized and eminently operable lesion for a considerable time. The method is not quite infallible, but the margin of error is very small. (Fig. 4.)

*X-rays.*—Chest surgeons should and do interpret their own X-rays. The only rational foundation for X-ray interpretation is the repeated correlation, at every possible opportunity, of radiological appearances with clinical, endoscopic, operative and autopsy findings. The opportunity to see inside more and more chests has led to greater circumspection in some erstwhile popular radiological diagnoses, such as unresolved pneumonia, interlobar effusion, and pulmonary fibrosis. No attempt will be made to discuss X-ray appearances in detail, but four ancillary methods of X-ray examination which are playing an important role in chest diagnosis call for comment.

First, lipiodol bronchography. The surgical treatment of bronchiectasis or a chronic lung abscess by modern methods of lobar

### PLATE I

FIG. 1.

Male, aged 31, treated by artificial pneumothorax for pulmonary tuberculosis for three years on account of collapse and cavitation in right upper lobe. Bronchoscopy revealed a bronchial adenoma obstructing the bronchus to this lobe. Total pneumonectomy performed.

FIG. 2.

Girl, aged 5, treated for hysteria from age of 1, on account of vomiting. Oesophagoscopy revealed an extensive stricture of the lower half of the oesophagus due to peptic ulceration of the mucosa. Partial oesophagectomy and oesophago-gastric anastomosis performed.

### PLATE II

FIG. 3.

Female, aged 37, diagnosed as a case of Liebman Sacks disease as a result of lung biopsy which revealed the characteristic histological changes in the branches of the pulmonary artery. Spontaneous recovery.

FIG. 4.

Male, aged 54; diagnosis of early carcinoma of left dorsal lobe made on exploratory thoracotomy. The growth was only 1 centimeter in diameter. Total pneumonectomy performed.

### PLATE III

FIG. 5.

Female, aged 24, complaining of repeated haemoptysis. Bronchogram revealed segmental bronchiectasis of lingular process of left upper lobe and anterior basic segment of left lower lobe.

FIG. 6.

Male, aged 34, referred as a case of mediastinal tumour and diagnosed as achalasia of the cardia on barium swallow examination. Heller operation performed.

PLATE IV

FIG. 7.

Male, aged 47, bronchogenic carcinoma of the peripheral type discovered on routine mass radiography. Total pneumonectomy performed.

FIG. 8.

Boy, aged 7, with extreme cyanosis. Screening revealed absence of pulsation in pulmonary vessels typical of congenital pulmonary stenosis. Cyanosis relieved by Blalock operation.

or segmental pulmonary resection with removal of all the diseased segments, and only the diseased segments, demands an exact diagnosis of the full anatomical extent of the disease, which can only be supplied by faultless bronchography technique. Slovenly technique has in the past resulted in some important misconceptions concerning the nature and spread of bronchiectasis. (Fig. 5.)

Second, the barium swallow and meal should seldom be omitted in unravelling obscure lesions of the mediastinum. The retrograde meal, with the patient in the head-low position may be the only method of demonstrating hiatus hernias of the diaphragm and growths at the cardiac end of the stomach. (Fig. 6.)

Third, tomography, the most recent refinement in X-ray technique, is destined to play an increasingly important role in the investigation of mediastinal masses, hilar glandular enlargement, tuberculous infiltration, and obscure intra-pulmonary disease. In tuberculosis it bids fair to revolutionize our conception of the incidence of cavitation. Tomographic examination of apparently minimal quiescent lesions in the better lung of cases proposed for thoracoplasty proves that cavitation and active infiltration are present in a large percentage of these so-called quiescent lesions. Some of the less satisfactory results of this operation can now be explained in the light of this additional insight into pulmonary pathology.

The Mass Miniature Radiography Unit is becoming more and more the "receptionist" to the thoracic unit of the future. Not only has thoracic disease been detected and diagnosed before the appearance of symptoms, but in many cases patients who are already aware that all is not well in the chest are first referred to the Thoracic Unit following routine mass radiography, when many months might have elapsed before the patient considered his symptoms sufficiently severe to warrant a visit to his doctor, by which time the disease might have progressed to a hopelessly inoperable stage. (Fig. 7.)

*Cancer.*—One of the most difficult problems that confronts the thoracic surgeon at the moment is the early diagnosis of cancer of the lung. This disease is diagnosable at an earlier stage and consequently more amenable to successful surgical treatment than any other form of internal malignant disease. It needs only a very small growth to obstruct a branch bronchus, and this bronchial obstruction produces symptoms which, in the majority of cases, are sufficiently severe to take the patient to his doctor. But at this stage the diagnosis can only be made on X-ray examination. Physical signs and the classical textbook symptoms, such as haemoptysis, dyspnoea and loss of weight, may be absent. Urgency is the key-note of the investigation of the patient suspected of having cancer of the lung, and the old policy of "wait and see"

must be abandoned for evermore. In 60 per cent. of the proved cases of carcinoma of the lung seen at this unit the initial symptoms were acute pain in the chest, associated with dry cough and pyrexia, usually diagnosed by the general practitioner as " bronchitis and pleurisy." These symptoms, which probably mark the onset of bronchial obstruction, usually disappear within three weeks, and not until six to nine months later do the classical textbook symptoms begin to appear. A localized " wheeze " in the chest is another important early symptom.

Carcinoma of the lung unassociated with obstruction of the larger or medium bronchi, the peripheral type of growth, may cause symptoms frequently and mistakenly assigned to other causes. Patients with pulmonary osteoarthropathy of the acute type have been treated for rheumatoid arthritis ; others with pain due to direct involvement of the chest wall for arthritis of the shoulder joint, gall stones, or " pleurodynia." Early pleural involvement may cause pain in the absence of X-ray changes, and there is the classic case of the man who was treated successively for pleurodynia, arthritis of the spine, schizophrenia, and tuberculosis because an original X-ray of the chest was said to have shown no abnormality ; two years too late the diagnosis of carcinoma was established by thoracoscopy and pleural biopsy. Most sanatoria have a few cases of carcinoma languishing in their wards and the rare association of carcinoma and tuberculosis almost invariably leads to neglect of the former condition. Hoarseness, swelling of the face due to caval obstruction, and weakness of the hand are sometimes the earliest symptoms caused by the spread of the growth to contiguous structures.

The diagnostic responsibilities of the thoracic unit are increasing. Cardiac surgery is now emerging from the purely experimental phase and is becoming established on a sound clinical basis. The progress already made in the technique of cardiac surgery demands a parallel advance in the application of methods of assessing normal and abnormal cardio-respiratory physiology. The thoracic surgeon called upon to extend his indications for operation to patients gravely disabled by serious congenital and acquired defects with their attendant aberrations of physiology must have at his disposal facilities for the accurate assessment of the degree of abnormality and its possible response to operative treatment. Such procedures as cardio-angiography, cardiac catheterization, blood gas analysis, and broncho-spirometry for the measurement of differential lung function must become as readily available as bronchoscopy and tomography are to-day. No thoracic unit will be complete without a fully equipped physiological laboratory. (Fig. 8.)

## Figures and Tables

**Fig. 1. f1:**
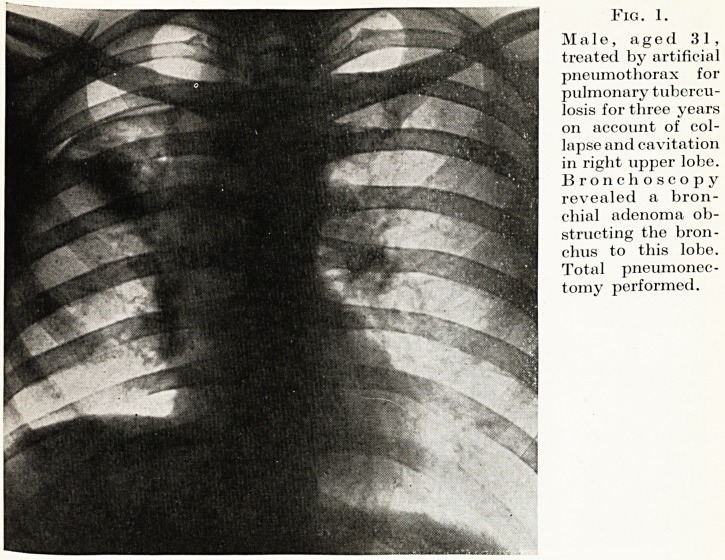


**Fig. 2. f2:**
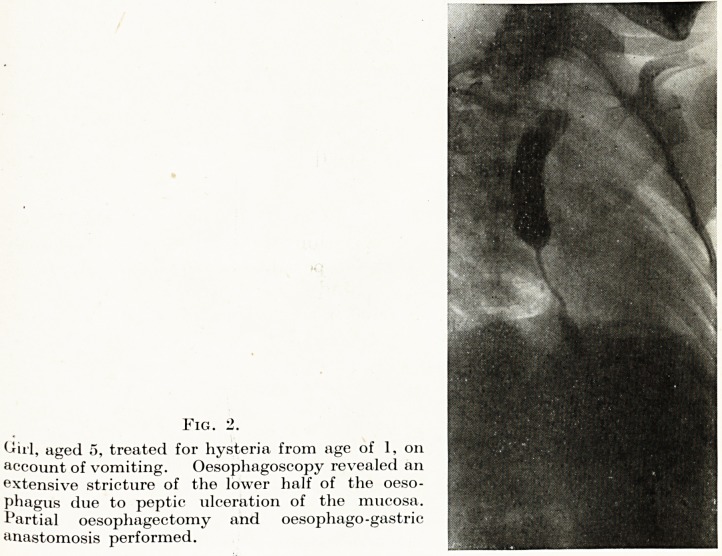


**Fig. 3. f3:**
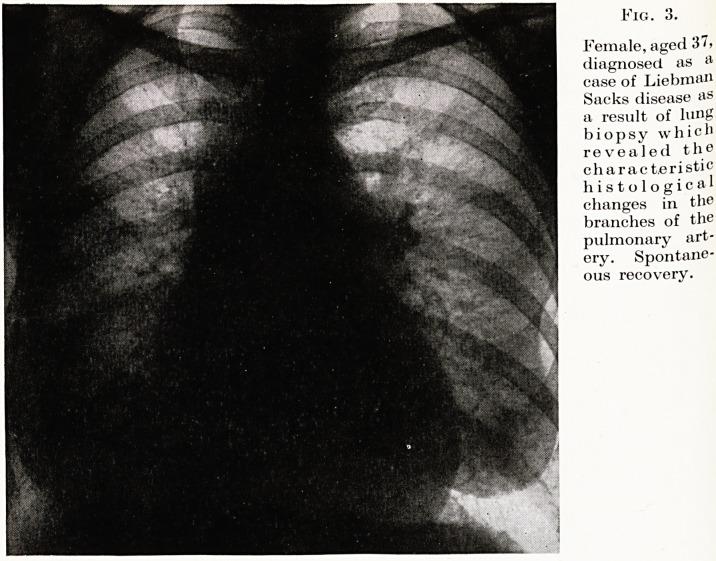


**Fig. 4. f4:**
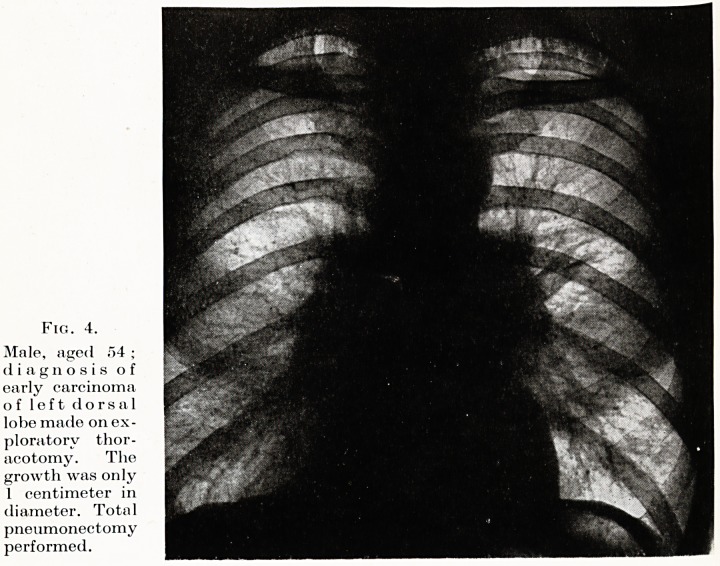


**Fig. 5. f5:**
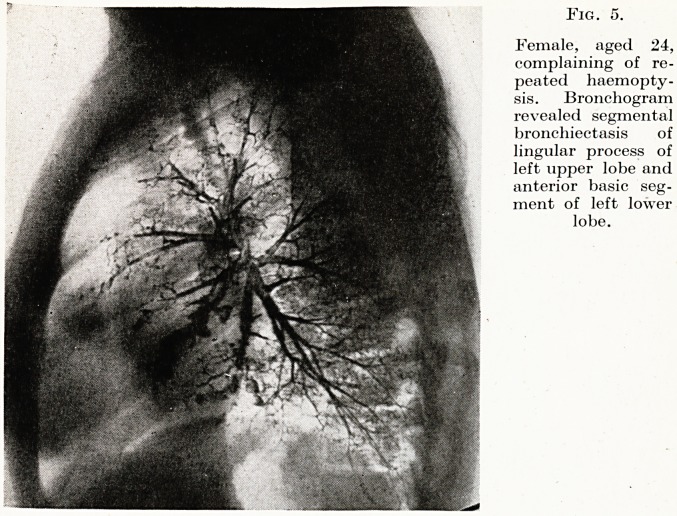


**Fig. 6. f6:**
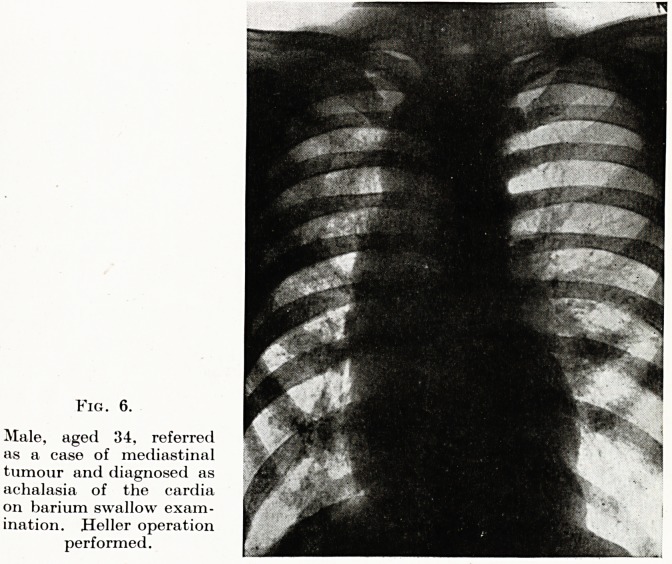


**Fig. 7. f7:**
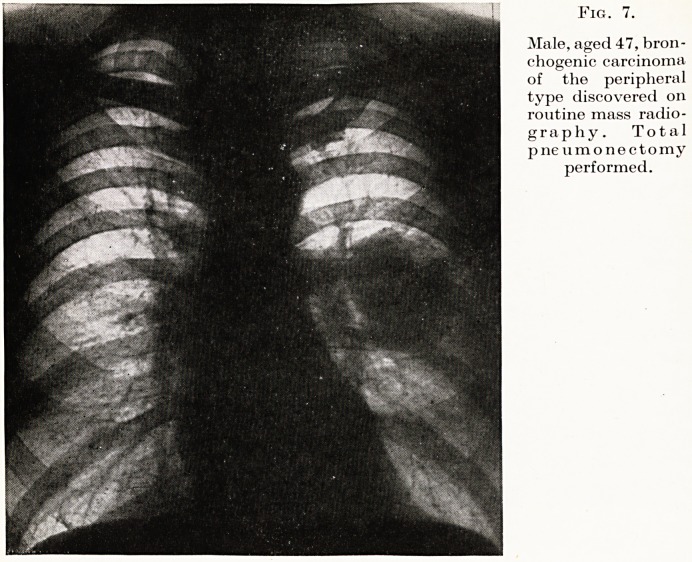


**Fig. 8. f8:**